# Management of Gold Alloys

**Published:** 1859-03

**Authors:** 


### Original Essays and Communications.

### MANAGEMENT OF GOLD ALLOYS.

BY GEO. WATT.

We have been frequently solicited, by some of our young professional friends, to insert in the "*Register,*" a few practical remarks on the treatment of gold scraps, and filings, in the Dental laboratory. In complying with this request we will endeavor to be understood, without attempting to be *scientific* or minute. Various processes and manipulations may be resorted to successfully, and almost every dentist has his favorites, hence, many may think our suggestions are not the best that might be given, and this opinion is very likely to prove correct.

The appropriate metals for reducing gold to the standard required for dental purposes, as all know, are silver, copper, and platinum. It is sometimes desirable to raise the standard of gold alloyed with these, or to remove them entirely from the mass. But the principle trouble with scraps and filings arises from the presence of what are called base metals. Of these, iron, tin, lead, and zinc are those most frequently met with. Any of these, in appreciable quantity, renders the gold brittle, and unfit for use, yet they differ somewhat in their influence on it. Tin causes less change of color than the others, but it forms a more brittle alloy with gold than any of them. The alloy of gold and lead is darker colored than that formed with tin, and is nearly as brittle. Zinc seems to have less affinity for gold than either tin or lead, and, hence, it appears to be unequally distributed through the alloy, and the consequence is that some parts of the ingot is far more brittle than others. If the attempt to roll it into plate be made while it is in this condition, some parts of it may manifest a good degree of plasticity, or malleability, while small spots will crack, causing the plate to be full of holes. The presence of platinum imparts to the alloy an exceedingly dull color, and it increases its hardness and elasticity, but does not render it brittle. To sum up these points ;—when the alloy is very brittle, while the color is rather bright, we may infer that tin is present ; if the color be very dull, while the alloy is tough and elastic, platinum may be suspected ; if the mass be both brittle and dull colored, lead is probably present, and if the brittleness be confined to limited spots, the presence of zinc is indicated.

In all our remarks thus far, we have taken it for granted that the base metals are present in only minute proportions, that gold greatly predominates over them. We are endeavoring to describe such alloys as the mechanical dentist is liable to meet with after taking what he supposes to be due care to prevent their formation. He will often find alloys not possessing any of the specific characters described above, for he may have several base metals combined with his gold at the same time.

Before noticing the method of separating gold and platinum, and the mode of obtaining pure gold, we will speak of the "dry process" for separating minute particles of base metals from dentists' gold. The scraps are supposed to be under treatment, and are known to be 18 or 20 carats fine, but in handling a trace of one or more of base metals is melted with them, and the gold is rendered unfit for use.

Now, if when the alloy is in a liquid state, some element be brought in contact with it which, by virtue of its affinity for the base metal, is capable of separating it from the gold, our end is accomplished. Whatever element be used, it must, of course, have a stronger affinity for the base metal than for the gold. And, fortunately, we are at no loss here, for gold has but little affinity for any of the non-metallic elements.

The only elements relied on, in the process under consideration, are oxygen, chlorine, and sulphur. The base metal is to be separated by converting it into an oxyde, a chloride, or a sulphuret. Without being considered responsible for the etymology used, we will speak of the three processes under the titles of *oxydation*, *chlorination* and *sulphuration*.

If we attempt to purify our gold, then, by oxydation, we want some compound containing oxygen, which is decomposed by heat. A variety of substances might be used, but nitrate of potash will usually accomplish all that can be obtained by this process. This salt is readily decomposed by heat, and the decomposition results in the liberation of oxygen. This may be more clearly understood by a glance at the formula representing its composition;—$KO.\ NO_5$ For sake of the non-chemical reader, I may be allowed to remark that K. is the symbol of potassium, O. that of oxygen, and N. that of nitrogen. (Potassium being formerly called *Kalium*, explains the retention of the symbol K.) Potassium and oxygen uniting form potash; and nitric acid, $NO_5$, uniting with this, forms nitrate of potash, which is the *nitre* or *salt petre* of commerce. Of the three elements composing this salt, oxygen is the only one that can act on the base metal, for nitrogen has but little affinity for any thing, and potassium has none for this class of metals. But oxygen is abundantly liberated as the nitrate is converted into a hypo-nitrite, thus:—

$$KO.\ NO_5 = KO.\ NO_3 + O_2.$$

Concluding, then, that this salt can do nothing but oxydate, we can readily understand how and when to use it.

When our brittle alloy is melted, a small quantity of the nitrate should be dropped into the crucible, without taking it from the fire; and, if thought necessary, additional quantities may be used till purification is effected. The roasting should be continued for some time, and it is often necessary to pour, and resume the process in a clean crucible, as the slag sometimes prevents the free access of the oxygen to the alloy. By a little practice the manipulator will learn the various minutiæ of the process.

The process of oxydation of course is not adapted to the separation of a metal from gold, which has but little affinity for oxygen. We mention this only because we have known dentists to roast their alloys with salt petre, for hours in succession, in an endeavor to separate platinum from gold. As neither metal is oxydated under such circumstances, the effort is simply a waste of time and fuel.

The process of oxydation, then, is to be resorted to when the base metal is readily oxydized. But if it have but little affinity for oxygen, then we should inquire into its affinities for chlorine and sulphur. Thus, if it be tin, for example, we could remove it better by chlorination than by oxydation.

Chlorination should always be resorted to when the base metal has a stronger affinity for chlorine than for oxygen or sulphur. Any compound which yields chlorine by the application of heat, and at the same time introduces no troublesome material into the alloy will answer the purpose. Some chlorides are not readily decomposed. For example, common salt, which is chloride of sodium, sustains a high heat without decomposition, and, consequently will not answer our purpose. The compound which we prefer, is the chloride of mercury, commonly called corrosive sublimate. It has several advantages over most other chlorides. It yields chlorine readily by the application of heat; and, at the same time, the mercury, instead of uniting with the gold, is driven off in the form of vapor. It may be used as directed for nitrate of potash, in the process of oxydation; but smaller quantities should be added at once, to prevent waste; and, indeed, smaller quantities will answer the purpose.

The well-known "sal ammoniac" is used for the same purpose, and in about the same way. This salt is the hydrochlorate of ammonia; (or, possibly, chloride of ammonium), and it yields chlorine by decomposition with heat.

Of course it is not practicable to separate, by this process, two metals which differ but little in their affinity for chlorine. Those who try to separate gold and platinum in this way, will, accordingly, fail. We have known some to roast, first with saltpetre, and then with corrosive sublimate, and then become disheartened, because their plate, though sufficiently tough, was still of a bad color. More than once, such gold has been sent to us to be refined, accompanied with a long, graphic history of the tortures it had endured, and the obstinacy with which it resisted refinement by roasting.

To remove the base metals, sulphur is sometimes dropped into the crucible after the alloy is melted, the manipulations being about the same as in oxydation with nitrate of potash. But, as all elements act with greater energy in their nascent state, that is, just as they are being liberated from a compound, if a sulphuret which is readily decomposed by heat be used, the process of sulphuration will be more active. In selecting a sulphuret, it is desirable to choose one which will not be the means of introducing a troublesome ingredient into the gold. For example, if a sulphuret of zinc were used for the removal of lead, we would gain little or nothing; as, instead of an alloy of gold and lead, we would have one of gold and zinc.

The native sulphuret of antimony is, perhaps, the most convenient of the sulphurets. This is a sesqui-sulphuret, and is readily obtained in most drug stores. It is desirable to resort to sulphuration only when the alloy is rendered very coarse by the presence of base metals. The alloy should be melted in a large crucible, and about twice its weight of the native sulphuret should be added, a small quantity at a time; and the roasting should be prolonged in proportion to the quantity of base metal present. As the sulphuret is decomposed, the sulphur unites with the base metals, converting them into sulphurets, and the antimony unites with the gold, forming a leaden colored alloy. The antimony is to be expelled by melting the alloy in a clean crucible and forcing a current of air on its surface, with a bellows. The antimony is oxydized, and passes off in vapor. The air should not be applied too rapidly at first, as the rapid escape of the oxyd of antimony might carry off portions of the gold with it. A distinctly visible escape of vapor is evidence of sufficiently rapid oxydation. As the antimony escapes, the melting point of the alloy raises. The force of the fire must, consequently, be increased.

We conclude, then, that, if lead be the contaminating metal, the oxydating process is the preferable one. Nitrate of potash will, therefore, answer the purpose. It is important to use clean crucibles; and to change for fresh ones, if a prolonged roasting is necessary. Zinc may be separated by either oxydation or chlorination, without much difficulty. Tin is much more readily removed by the latter, than by the former process. Indeed, it is oxydized with such difficulty, that, when protected by the gold, it is almost impracticable to remove it entirely by oxydation. Iron may be separated by any of the three processes.

In many cases, tin, lead, and zinc, are all present. If persuaded of this, we usually begin with oxydation. If after reasonable roasting with nitrate of potash, the alloy is still brittle, we infer that the difficulty arises from the presence of tin, and we use the chloride of mercury (corrosive sublimate).

But if the alloy be very impure, we prefer the wet method which we will notice directly, and by which we obtain pure gold, which may be reduced to the required standard.

The management of filings, with but slight modifications, is to be conducted on the same principle as that of scraps.

Particles of iron or steel derived from the file are usually present. These may be readily removed by the magnet before commencing other treatment. By digesting the filings with nitric acid, in a glass or porcelain vessel, the base metals are oxydized, and converted into nitrates, which are soluble, and may then be removed by washing with water. Metals, when minutely divided, do not always flow readily together when melted in a crucible. The tendency is to the formation of minute globules, which remain separated from the mass. A good method of overcoming this is to use, as a flux, the dry carbonate of potash, the *sal tartar* of the shops. If the crucible be lined with borax, and the filings be added, after digesting in nitric acid, the addition of an equal bulk of the carbonate will answer all purposes. By raising the heat to the melting point of the filings they will then run together into a single mass, which may be refined if necessary, as above described for the treatment of scraps.

It is scarcely necessary to mention the use of borax as a flux, in this connection. When the gold is of the proper quality for dental purposes, the addition of borax protects it from oxydation, which might, when unprotected, lessen the relative quantities of copper and silver in the alloy. And, as all metallic oxyds are soluble by heat in this salt, it insures clean metallic surfaces for contact, and, consequently, a ready flowing together of the melted metals.

When the alloy is composed of metals differing but little in their affinities for oxygen, chlorine, etc., we resort, as stated above, to one of the "wet methods." And, in this connection, we will only describe the one which we consider the most convenient and effectual for the practical dentist. It is effectual in all cases, as it always gives us pure gold.

Let us, then, suppose that our gold alloy has become contaminated with platinum to such extent that the color and elasticity of the plate is objectionable. The alloy should be dissolved in nitro-hydrochloric acid, called *aqua regia.* The best proportions for aqua regia are three parts of hydrochloric acid to one of nitric. If the acids are at all good, four ounces of the aqua regia will be an abundance for an ounce of the alloy. The advantage of using the acids in the proportion of three to one, instead of two to one, as directed in most of the text books, is that when the solution is completed there is but little, if any excess of nitric acid. If the acids be "chemically pure," four parts of the hydro-chloric, to one of the nitric, produces still better results.

By this process the metals are all converted into chlorides; and, as the chloride of silver is insoluble, and has a greater specific gravity than the liquid, it is found, as a grayish white powder, at the bottom of the vessel. The chlorides of the other metals, being soluble, remain in solution. By washing and pouring off, allowing the chloride of silver time to settle to the bottom, the solution may be entirely separated from it. The object is now to precipitate the gold while the others remain in solution. This precipitation may be effected by any one of several different agents, but we will mention only the proto-sulphate of iron.

This salt is the common green copperas of the shops, and, as it is always cheap and readily obtained, we need look no further. It should be dissolved in clean rain-water, and the solution should be filtered, and allowed to settle till perfectly clear. Then it is to be added gradually to the gold solution, as long as a precipitate is found, and even longer, as an excess will the better insure the precipitation of all the gold. The gold thus precipitated is a brown powder, having none of the appearances of gold in its ordinary state. The solution should now be filtered, or the gold should be allowed to settle to the bottom, where it may be washed after pouring off the solution. It is better to filter than decant, in this case, as, frequently, particles of the gold float on the surface, and would be lost in the washings, by the latter process.

Minute traces of iron may adhere to the gold thus precipitated. These can be removed by digesting the gold in dilute sulphuric acid; and, when the process is properly conducted, thus far, the result is PURE GOLD, which may be melted, under carbonate of potash, in a crucible lined with borax, and reduced to the desired carat.

The chloride of silver may be decomposed by mixing it with granulated zinc, and digesting it in dilute sulphuric acid. The hydrogen thus generated takes the chlorine to form hydro-chloric acid; and pure silver is left in a fine powder, which may be melted under borax or carbonate of potash. Or, if the carbonate of potash be first fused in a large crucible, and the chloride gradually added, it is decomposed; and when the heat is raised to the melting point of silver, the metal flows to the bottom of the crucible, where it should be allowed to cool, as it would not readily pour from the crucible under such circumstances.

The separation of the platinum from the solution will probably be noticed in some other connection.

